# Evaluation of the antibacterial effects and mechanism of Plantaricin 149 from *Lactobacillus plantarum* NRIC 149 on the peri-implantitis pathogens

**DOI:** 10.1038/s41598-021-00497-y

**Published:** 2021-10-25

**Authors:** Xiaolong Lin, Jiajia Xu, Zhiwei Shi, Yuedan Xu, Tao Fu, Ling Zhang, Fuming He

**Affiliations:** Department of Prosthodontics, Stomatology Hospital, School of Stomatology, Zhejiang University School of Medicine, Zhejiang Provincial Clinical Research Center for Oral Diseases, Key Laboratory of Oral Biomedical Research of Zhejiang Province, Cancer Center of Zhejiang University, Hangzhou, 310006 China

**Keywords:** Microbiology, Medical research

## Abstract

Peri-implantitis is a common reversible disease after tooth implantation, caused by a variety of pathogenic microorganisms. Based on non-surgical or surgical treatment principles, supplementation by local or systemic drugs might enhance treatment efficacy. *Porphyromonas gingivalis* (Pg) (ATCC 33,277) and *Prevotella intermedius* (Pi) (ATCC 25,611) were used as test strains. The effects of Pln 149 on the biofilm formation and growth of four periodontal pathogens were evaluated by RT-PCR, fluorescence microscopy, and scanning electron microscopy. The antibacterial mechanism was tested by the patch-clamp technique. The cytotoxicity of Pln 149 (125 µg/ml) to bone marrow stromal cell (BMSC) was assessed using an MTT assay. Pln 149 exhibited significant inhibitory effects on Pg and Pi (P < 0.05), with significant differences in the biofilm images of fluorescence microscope and scanning electron microscope (P < 0.05). Pln 149 could change the sodium channel currents and exerted no cytotoxicity on bone marrow stromal cell. Pln 149 could inhibit the biofilm formation and growth of periodontal pathogens. Considering the absence of antimicrobial resistance and cytotoxicity, we suggest that the Pln 149 from *Lactobacillus plantarum* 149 might be a promising option for managing peri-implantitis.

## Introduction

Peri-implantitis is a common reversible disease after tooth implantation, which occurs in oral soft tissues around the implant. Peri-implantitis is caused by a variety of pathogenic microorganisms and often requires treatment in various ways^1^. Non-surgical or surgical treatment, supplemented by local or systemic drugs, might improve treatment efficacy^2^. Removing and controlling biofilms on the implant are the key to the treatment of the inflammation. However, due to differences between implant materials and natural tooth structures, it is more difficult to remove dental plaque from implant surfaces^3^. Therefore, the auxiliary role of drug therapy becomes more important in the treatment of peri-implantitis.

Antibacterial peptides are small peptides, < 100 amino acids long, with broad-spectrum antibiotic properties. They are widely available in nature, both in plants and animals^4^. *Lactobacillus plantarum* is a probiotic that can produce organic acid, hydrogen peroxide, and diacetyl; it can also produce a small molecular protein with bacteriostatic activity, called bacteriocin^5^. Compared with traditional antibiotics, the antibacterial peptides have more advantages, such as no residues, no resistance, and easy protease degradation in the human gut, and have become a new option for treating infectious diseases^6^. Plantaricin 149 (Pln149), is a cationic antimicrobial peptide produced by *Lactobacillus plantarum* NRIC 149 that has been identified as a bacteriocin with a molecule weight of 2.2 kDa and was also heat-stable and active at pHs ranging from 4.0–7.0. Earlier studies have shown that Pln 149 exerts a strong antibacterial effect on common clinical pathogens^7^. Our previous study also showed that the peptide secreted by *L. plantarum* exerted strong inhibitory effects on *S. mutans and P. gingivalis*. Therefore, the use of Pln 149 from *Lactobacillus plantarum* 149 in the treatment of peri-implantitis seems relevant.

In this study, we aim to study the antibacterial effect of Pln 149 on the peri-implantitis pathogen in vitro and test the antimicrobial resistance and cytotoxicity. For that, we determined the bacterial count in the biofilm and used confocal laser scanning microscopy and transmission electron microscopy to verify the effects of Pln 149 in the treatment of peri-implantitis. Meanwhile, the antimicrobial resistance on *Staphylococcus aureus* and cytotoxicity on osteoblasts were tested to verify the clinical value of Pln 149.

## Methods

### Strains and culture conditions

*Porphyromonas gingivalis* (Pg) (ATCC 33,277), *Staphylococcus aureus* (ATCC 29,213), and *Prevotella intermedius* (Pi) (ATCC 25,611) were purchased from ATCC. Cultures of Pg and Pi were grown overnight in brain heart infusion broth (BHI) (Difco, USA) under anaerobic conditions (80% N_2_, 10% CO_2_, and 10% H_2_) at 37 °C in an anaerobic chamber. *S. aureus* was grown overnight in BHI in a microaerophilic environment (5% CO_2_). Then, the bacterial suspensions were centrifuged at 2000 rpm/min for 5 min, and the final concentrations of the cultures were adjusted to 0.5 × 10^5^ CFU/mL using a spectrophotometer.

### Peptide

Pln 149 (YSLQM GATAI KQVKK LFKKK GG) was synthesized by Shanghai Apeptide Co. Ltd (Shanghai, China). The peptide was purified by high-performance liquid chromatography, and its identity was verified by SDS-PAGE. The purity of Pln 149 (> 95%) and mass were confirmed by electrospray ionization mass spectrometry.

### Minimum inhibitory and minimum bactericidal concentration

The minimum inhibitory (MIC) and minimum bactericidal (MBC) concentrations of Pln 149 were determined for all the strains using a modified version of the Clinical Laboratory and Standard Institute (CLSI) broth microdilution method as described previously^8^. Bacterial suspensions were prepared to a final concentration of 5 × 10^5^ CFU/mL in BHI and added to wells in a sterile 96-well microtiter plate containing two-fold dilutions of Pln 149. The blood plate was incubated at 37 °C for 24 h. The MIC was set as the lowest concentration of peptides that reduced bacterial growth by ≥ 90%, while the MBC was set as the lowest concentration of peptides that reduced bacterial growth by > 99.99% after the enumeration of viable bacteria by plate counts compared to bacteria grown without antimicrobial peptides.

### Biofilm formation

Biofilm formation was assessed using a previously published method^9^. Pg and Pi culture were incubated in BHI broth and grown under an anaerobic aerophilic condition for 24 h at 37 °C. The cells were washed three times with a saline solution and then adjusted with saline to 0.5 × 10^5^ CFU/mL. Individual sterile petri dishes were filled with 9.9 ml BHI broth by a concentration of 100 µg /ml Pln 149. Then a sterile saliva-coated titanium plate was added to each one and individual or mixed 100 µL Pg and Pi suspensions were mixed in the dishes. As negative and positive controls, PBS and Chlorhexidine (CHX) suspensions (100 µg/ml) were used, respectively. All the Petri titanium plates were incubated in anaerobic conditions (80% N_2_, 10% CO_2_, and 10% H_2_) at 37 °C in an anaerobic chamber.

### The analysis of biofilm formation on titanium by a quantitative real-time polymerase chain reaction

During the 24 h of biofilm formation, the titanium pieces were placed in 1 mL of PBS buffer and vortexed for 90 s. The quantitative real-time polymerase chain reaction was conducted by the method of Choi EJ^10^. The culture was then diluted in PBS to 10^–3^. Finally, bacterial RNA from 100 µl of the suspension was prepared and analyzed by previously described methods. Real-time PCR was performed in triplicate in a 10 µl reaction volume containing 1 × SYBR Green Master Mix (DBI, China), 100 nM specific primer, and 50 ng template DNA on an ABI PRISM 7900HT system (Applied Biosystems Inc, USA) in 384-well PCR plates, the qPCR Primers used in this study are shown in Table 1.

**Table 1:** qPCR Primers used in this study.

Species	Sequence(5' to 3')	Target gene	Amplicon size(bp)
*P.gingival*	F:GGAAGAGAAGACCGTAGCACAAGGA R: GAGTAGGCGAAACGTCCATCAGGTC	*rpoB*	143
*P.intermedia*	F:CCACATATGGCATCTGACGTG R:TCAATCTGCACGCTACTTGG	*rpoB*	117
*Total Bacteria*	F:CCATGAAGTCGGAATCGCTAG R:GCTTGACGGGCGGTGT	*16S rRNA*	89

### Laser confocal microscopy

After a total of 24 h of biofilm formation, titanium was washed with 10 mL of phosphate-buffered saline (PBS) to remove unattached cells. The titanium were then fixed with 2.5% glutaraldehyde at 4 °C for 1 h. After fixation, the titanium were stained with a 0.01% acridine orange solution and washed three times with PBS. Any resulting biofilm was observed under a Zeiss LSM 710 laser (Zeiss, Germany) confocal microscope equipped with an argon laser with an excitation wavelength of 488 nm. All the images were captured and saved using Zeiss ZEN 2010 software (version 6.0; Zeiss, Germany)^11^.

### Electron microscopy

Scanning electron microscopy (SEM) was used by previous methods to visualize the damage to biofilm caused by Pln 149^12^. The Pg and Pi mixed biofilms were formed on titanium and washed with PBS. The biofilms were then treated with Pln 149 for 24 h, followed by fixation in 2.5% glutaraldehyde in 0.1-M phosphate buffer at pH 7.3. The dried samples were examined under the scanning electron microscope (NNS-450, FEI Company). Digital images were taken under the 5000 magnified visual field at 5kv.

### Planar lipid bilayer preparation and electrical measurements

The whole‐cell patch-clamp technique was conducted by the methods of Ghaderi^13^. The whole‐cell patch-clamp technique was employed at room temperature (23–25 °C) to measure ionic currents. Pipettes were pulled from glass capillaries that exhibited a resistance of 1–3 MΩ when filled with the pipette solution. The currents were measured in two sets of solutions, one designed to isolate the Na^+^ current and the other to measure K^+^ currents, as described below.

The primary cultured mouse dorsal root ganglion neurons (DRGs) were isolated and cultured according to the standard method. After 24 h, the whole-cell voltage-gated sodium and potassium currents were recorded and plotted the current–voltage curves. When sodium and potassium channel currents were recorded, standard extracellular fluid (NaCl 140 mmol/L, KCl 15 mmol/L, CaCl_2_ 2 mmol/L, MgC1_2_ 1 mmol/L, HEPES 10 mmol/L, Glucose 10 mmol/L) and electrode inner liquid (K-gluconate 140 mmol/L, KCl 5 mmol/L, MgC1_2_ 2 mmol/L, EGTA 10 mmol/L, K2-ATP 2 mmol/L, GTP 0. 1 mmol/L), The cells were clamped at − 80 mV and given a step voltage of 100 ms, + 10 mV, and gradually depolarised from − 80 mV to + 60 mV. Statistical values are given as means ± 95% confidence limits.

### MTT assay

The cytotoxicity of Pln 149 (125 µg/ml) to bone marrow stromal cell (BMSC) was assessed using an MTT cell proliferation kit (Roche Applied Science)^14^. The BMSC were incubated at 37 °C under 5% CO_2_ for 1, 2, and 3 days after cell inoculation as described previously. A 50-mL volume of MTT working solution was added to each well, and the mixture was incubated for another 4 h. Purple crystal formazan was observed around cells at × 40 magnification under a microscope. The cell medium was carefully removed, and then 100 mL of dimethyl sulfoxide was added to each well to dissolve formazan. After 15 min of incubation at 37 °C to completely dissolve formazan, the absorbance at 490 nm was measured on an enzyme-linked immunosorbent assay (ELISA) plate reader, and the results were expressed as optical density (OD) values.

### Drug resistance test

*S. aureus* was grown overnight in BHI in a microaerophilic environment (5% CO_2_). The Disc diffusion test was used for the common antibiotics sensitivity test of Pln 149 (30 µg) and Cefuroxime (30 µg)^15^. Then *S. aureus* was added in sublethal concentrations of Pln 149 and Cefuroxime (both 70 µg/ml) prepared by the BHI method in a microaerophilic environment for 12 h. The Disc diffusion test was tested after 0, 12, 24 and 48 times of culturing in the methods mentioned above. The inhibition zone diameters were determined with filter paper.

### Statistical analysis

All tests were repeated three times. The data were analyzed using GraphPad Prism 5.0. One-way ANOVA and Tukey multiple comparison tests were used for comparisons between different treatments. A P-value < 0.05 was considered statistically significant.

## Results

### Growth and biofilm inhibition of Pln 149 on Pg and Pi

**Figure 1:**
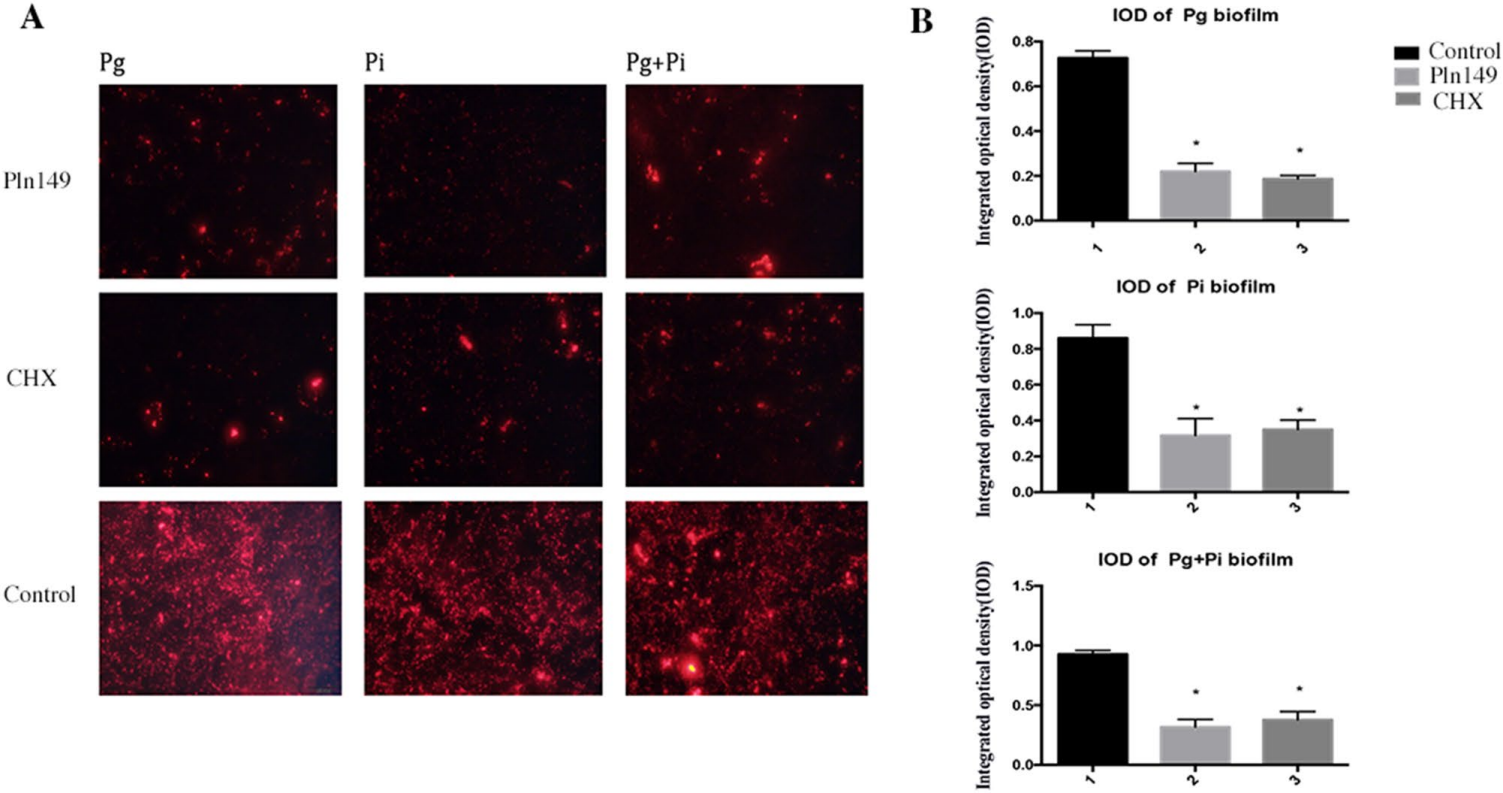
(**A**) Microscopic fluorescence images of biofilms on titanium-plate in blank control, Chlorhexidine (CHX, 100 µg /ml) -treated and Pln149-treated groups (100 µg /ml) after 24 h; (**B**) The integrated optical density (IOD) of biofilms on titanium-plate in blank control, CHX-treated (100 µg /ml) and Pln149-treated (100 µg/ml) groups after 24 h (mean ± SD, *P < 0.05).

**Figure 2:**
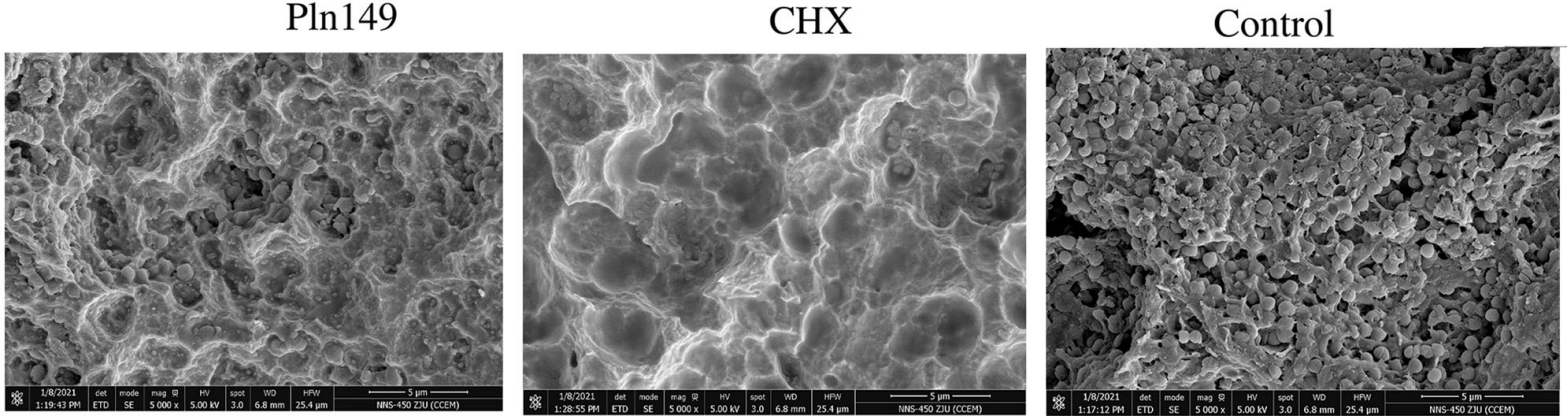
Scanning electron microscope of Pg and Pi mixed biofilms on titanium-plate in blank control, Chlorhexidine (CHX) -treated (100 µg /ml) and Pln149-treated (100 µg /ml) groups after 24 h.

Table 2 presents the results of MIC and MBC of Pln 149 on Pg and Pi. Pln 149 exerted strong antibacterial effects on biofilm formation on individual or mixed Pg and Pi. Figure 1 presents the fluorescence microscope images the IOD of biofim, Table 3 presents the results of RT-PCR of viable cells of *P.gingival* and *P.intermedia* on titanium plate, Pln 149 reduced the biofilm formation on individual or mixed Pg and Pi significantly (P < 0.05, Fig. 1, Table 3). Figure 2 presents the images of scanning electron microscope (SEM), which showed the cells of *P.gingival* and *P.intermedia* on titanium plate holes in Pln 149 groups were comparatively rare, the images were in constant with the RT-PCR results.

**Table 2:** Primary structure, molecular weight, MIC and MBC of Pln149 on *P.gingival, P.intermedia* and *S.aureus* ; *and* MIC and MBC of Cefuroxime on *S.aureus.*

Antibiotics	Sequence	MW	*P.gingival*		*P.intermedia*		*S.aureus*	
			MIC	MBC	MIC	MBC	MIC	MBC
Pln149	YSLQMGATAIKQVKKLFKKK GG	2130	125	125	100	100	78	78
Cefuroxime		424	30	15	20	20	80	80

Values are given in µg /ml.

**Table 3:** RT-PCR of viable cells of *P.gingival* and *P.intermedia* on titanium plate(, Data are expressed as the mean ± SD;* p < 0.05).

	Pg	Pi	Pg + Pi
Pln149	1.21 ± 0.28 × 10^7^/ml *	0.98 ± 0.33 × 10^7^/ml *	2.01 ± 0.42 × 10^7^/ml *
Chlorhexidine	1.01 ± 0.43 × 10^7^/ml*	0.78 ± 0.29 × 10^7^/ml *	0.95 ± 0.26 × 10^7^/ml *
Control	3.43 ± 0–89 × 10^7^/ml	3.39 ± 1.01 × 10^7^/ml	7.54 ± 2.78 × 10^7^/ml

### Whole cell patch clamp of Pln 149

**Figure 3:**
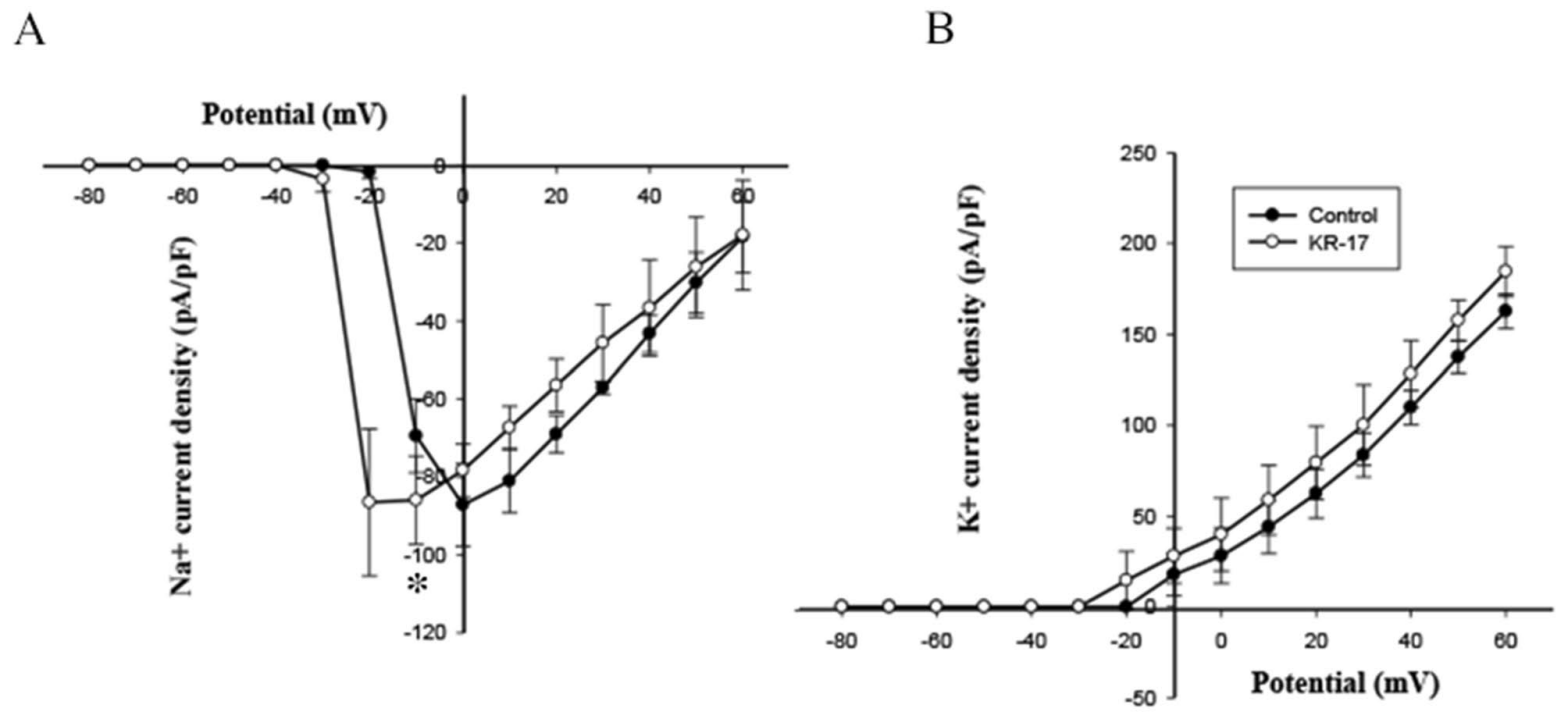
I-V curve of sodium (**A**) and potassium channel (**B**) currents on dorsal root ganglion neurons (DRGs) in control groups and Pln149-treated groups.

Whole cell patch clamp of Pln 149 were conducted at a final concentration of 125 µg/ml of Pln 149. Figure 3 presents the results. Pln 149 enhanced the sodium current at − 20 mM voltages (P < 0.05, Fig. 3). While the Pln 149 on the potassium curve moved to the left, the highest response changed slightly, with no significant difference (Fig. 3).

### MTT experiment analysis

**Figure 4:**
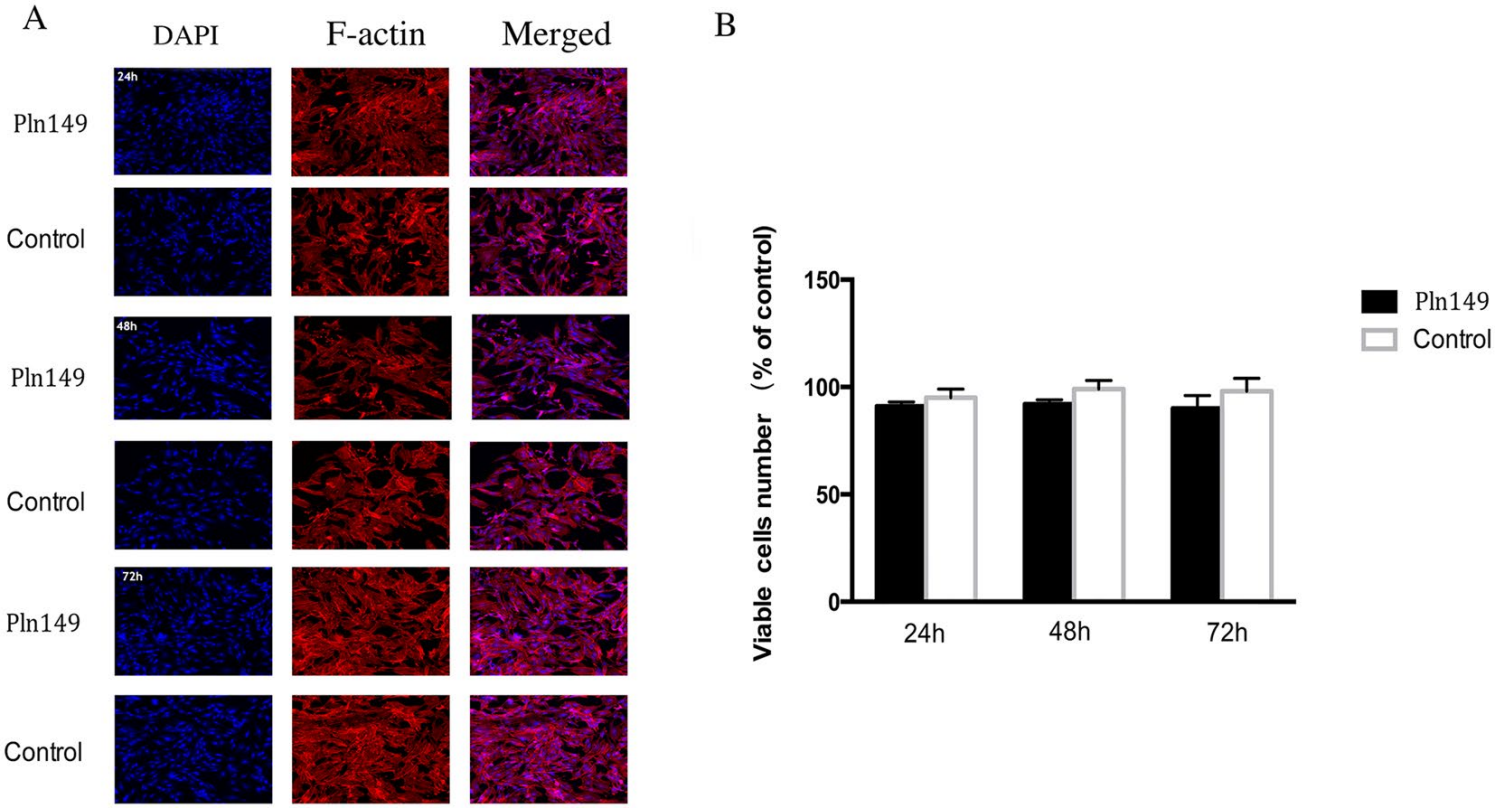
(**A**) Microscopic fluorescence images showing no morphological changes of the primary bone marrow stromal cell (BMSC) and BMSC cells treated with Pln149 for 24 h, 48 h, and 72 h. (**B**) The BMSC cell viability was measured by MTT assay. Values are expressed as mean and SD. *p < 0.05 compared with the control group.

As shown in Fig. 4A, after 24 h, 48 h and 72 h culture, the cell morphology of BMSC fluorescence stained by DAPI and F-actin showed no obvious changes. Meanwhile, there was no significant difference in cell viability or proliferation between the Pln 149 and control groups throughout the 24 h, 48 h and 72 h culture period (Fig. 4B).

### Drug resistance test

**Figure 5:**
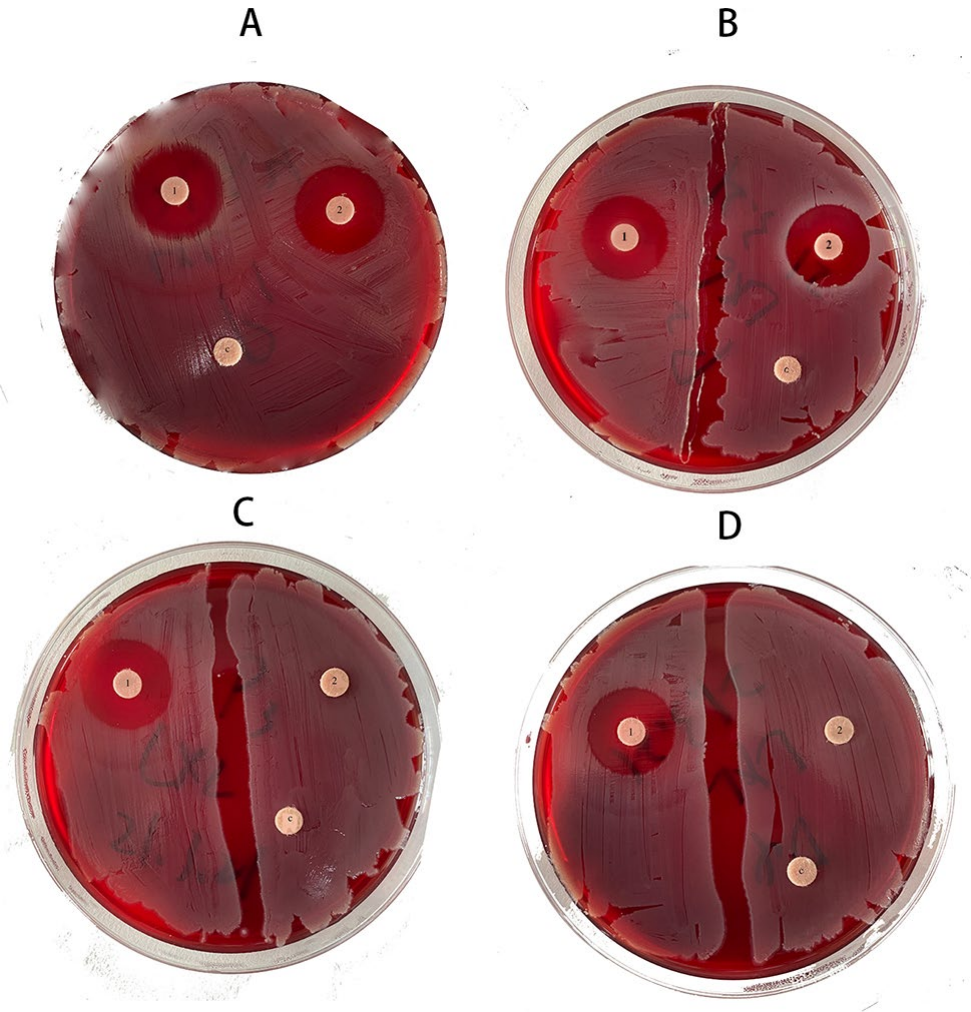
*S. aureus* after 12, 24, 48 times culture with sublethal concentrations of Cefuroxime and Pln149 using a 0.5 McFarland inoculum that were characterized by 100ug Cefuroxime and Pln149 disk diffusion test (**A **original strain; **B** *S. aureus* after 12 times culture; **C** *S. aureus* after 24 times culture; **D** *S. aureus* after 48 times culture; 1: Pln149 groups; 2: Cefuroxime groups; c: control groups).

As shown in Fig. 5, after 12, 24, and 48 times of culture, the inhibition zone diameters of Pln 149 showed no significant differences compared with original *S. aureus*. However, the inhibition zone diameters of Cefuroxime began to decrease after 24 times of culture, and with almost no antibacterial effects after 24 times of culture (Table 4).

**Table 4:** Antimicrobial activity of Pln149 (30 µg) and Cefuroxime (30 µg) against *S.aureus* (after 12,24,and 48 times culture under 70 µg /ml sublethal concentrations of Pln149 and Cefuroxime) using paper disk diffusion method.

Antibiotics	0	12	24	48
Pln149	24.09 ± 2.37	23.78 ± 2.98	23.09 ± 2.18	23.01 ± 1.28
Cefuroxime	21.45 ± 3.98	18.78 ± 3.09	6.93 ± 0.78	0

Diameter of inhibitory zone < 7 mm considered as no antimicrobial activity. Values are mean diameter of inhibitory zone (mm) ± SD of five replicates.

## Discussion

Peri-implant disease is among the most important reasons for the failure of dental implants. Recent research showed that peri-implant mucositis and peri-implantitis (PI) prevalence rates were 64.6% and 8.9%, respectively^16^. Many researchers believe that bacteria can be transferred from the periodontitis and periarthritis areas to implant-surrounding tissues; therefore, microorganisms associated with PI are similar to those inducing periodontal and periapical diseases^17^. Compared with using common treatments (including plaque control, mechanical debridement, antibiotics, physiological saline solution, hydrogen peroxide flushing, surgical treatment, and laser treatment), the local or systemic release of antibiotics can eliminate periodontopathogens to a large extent to promote the healing of tissues surrounding implants with PI^18^. Since local or systemic delivery of antibiotics plays an essential role in the treatment of PI, finding effective antibiotics seems crucial. Pg and Pi are extremely susceptible to grow on the rough surface of the implant^19^, our present SEM study showed that Pln 149 could significantly reduce the number of Pg and Pi on titanium surface, consistent with previous studies^20^.

Many studies have already compared the biofilm microbial proteomics in PI and health around implants or periodontitis. Compared with periodontitis samples or healthy implants, the biofilm in PI-surrounding tissues mainly comprises Pg and Pi^21^. However, PI biofilm might be worse than the surrounding tissue of periodontitis because PI has a broader spectrum of microorganisms. According to the present study, antibiotics as adjunctive drugs of PI routine treatment can promote the resolution of inflammation in tissues surrounding implants^22^. Mechanical debridement and antibiotic therapy could eliminate a significant number of bacteria in biofilms. However, due to the surface characteristics of various implant systems and differences between the forms and surface treatments, it is difficult to remove biofilms from implant surfaces completely. Therefore, it is necessary to administer topical and systemic antibiotics, as PI conventional adjunctive treatment, to more thoroughly remove biofilms from the implant surfaces and microorganisms around the lesions.

The discovery of penicillin brought hope for the treatment of infectious diseases caused by pathogenic microorganisms, leading to the development of many antibiotics, with significant contributions to protecting human health^23^. However, the widespread use of these “traditional antibiotics” continuously gave rise to many new problems, such as drug-resistant strains. Therefore, researchers began to look for a new generation of antibacterial agents. Cefuroxime belongs to the second generation cephalosporins, the antibacterial effects against gram-negative bacteria is enhanced compared with the first generation cephalosporins. As shown in MIC and MBC tests, cefuroxime actually exerted stronger antibacterial effects against Pg and Pi compared with Pln 149, and they showed the same antibacterial effects against *S. aureus.* Researches showed that cefuroxime especially likely to develop drug resistance in *S. aureus* and *P. Aeruginosa*^24^, our results showed that Pln 149 was difficult to form drug resistance. The osseointegration of the implant which is the key to the success of dental implant is based on the establishment of bone-implant surface contact. Osteoblasts originated from bone marrow stem cells, play an important role in the secretion, mineralization and synthesis of bone matrix^25^, Pln 149 showed no cytotoxicity on BMSC and low levels of drug resistance which indicated that the use of Pln 149 for a longer period of time is safe and efficient.

Recent studies have found that some cationic peptides exhibit a broad-spectrum antimicrobial activity, resulting in no drug-resistant bacteria^26^. Studies on bacteriocin isolated from lactic acid bacteria (LAB) have confirmed that some bacteria in this group, such as *L. rhamnosus GG*, can produce bacteriocin, significantly impacting most gram-positive and some gram-negative bacteria^27^. Previous studies have shown that *Lactobacillus reuteri* could more effectively alleviate patients’ symptoms, improve the microbial environment around the implant, and exert a better therapeutic effect on PI^28^. The study of Jae-In Jung showed that *Lactobacillus plantarum* has great potential as a multifunctional oral health ingredient that inhibits biofilm formation and suppresses the alveolar bone loss associated with periodontitis^29^. However, the relevant studies have not indicated the substances and mechanisms involved. In the present study, Pg and Pi counts on the titanium plate decreased significantly, consistent with previous studies indicating that Pln 149 produced by probiotic *Lactobacillus plantarum* could positively impact PI treatment^30^. The antibacterial mechanism of traditional antibacterial drugs mainly includes inhibition of bacterial cell-wall biosynthesis, bacterial protein synthesis and bacterial DNA replication and repair^31^. However, eighty percent of bacterial infections are related to the formation of bacterial biofilm, traditional antibacterial drugs could not permeate into the bacterial biofilm. Compared with planktonic bacteria, bacterial biofilm is 10–1000 times more resistant to antibiotics, which is the main cause of current bacterial drug resistance^32^. Metabolic regulation between distant bacteria in biofilms may involve electrochemical communication^33^. Study showed that ion channels conduct long-range electrical signals within bacterial biofilm communities through spatially propagating waves of potassium. Our study showed that Pln 149 could change the sodium channels which indicated that Pln 149 could inhibited the biofilm formation by ion channel. Compared with traditional antibacterial drugs used in oral, Pln 149 exerted the strong anti-biofilm effects, low drug resistance, excellent biocompatibility, and the use of Pln 149 may provides new ideas for the treatment of peri-implantitis.

It is interesting to observe the antibacterial effect of Pln 149 on the growth of Pg and Pi; however, the implant in the oral cavity is inhabited by a highly diverse and dense population of bacteria, which form a complex ecosystem that is difficult to simulate in vitro. Therefore, studying the antibacterial effect of Pln 149 in vivo is still necessary to understand the role of Pln 149 in the PI treatment. We will provide PI patients with probiotic products to detect peri-implant micro-ecological changes using high-throughput sequencing technologies in future experiments.

In conclusion, Pln 149 could inhibit the growth of Pg and Pi in vitro, impacted the bacteria in the biofilm on titanium plates by changed the sodium channel currents, and exhibited safety and a good application value in PI treatment.

## Data Availability

The authors declare that all data and materials support published claims and comply with field standards.
